# AXL phosphorylates and up-regulates TNS2 and its implications in IRS-1-associated metabolism in cancer cells

**DOI:** 10.1186/s12929-018-0465-x

**Published:** 2018-11-12

**Authors:** Li-Chun Cheng, Yen-Lin Chen, An-Ning Cheng, Alan Yueh-Luen Lee, Chun-Yu Cho, Jhy-Shrian Huang, Shuang-En Chuang

**Affiliations:** 1 Graduate Institute of Life Sciences, National Defense Medical Center, Taipei, Taiwan; 2 Department of Pathology, Cardinal Tien Hospital, School of Medicine, Fu-Jen Catholic University, New Taipei City, Taiwan; 3 National Institute of Cancer Research, National Health Research Institutes, 35 Keyan Road, Zhunan, Miaoli County 350, Taiwan, Republic of China; 4 Department of Pediatrics, Kaohsiung Medical University Hospital, Kaohsiung, Taiwan

**Keywords:** Axl, Tensin2, C1-TEN, IRS1, PDK1, Glut4, Pancreatic cancer, Glucose metabolism

## Abstract

**Background:**

TNS2 is a focal adhesions protein and a binding partner for many proteins, including the receptor tyrosine kinase Axl. Although TNS2 can bind with Axl, the details of their interactions have not been elucidated. TNS2 is involved in IRS-1 signaling pathway. In this study, we confirmed the relationship between TNS2 expression and the expression of Axl, IRS-1, PDK1 and Glut4 in pancreatic cancer patients.

**Methods:**

The expression levels of TNS2, Axl, IRS-1, PDK1 and Glut4 in human cancer cells were measured by Western blot and/or IP-Western blot assays. Paired samples of pancreatic cancer and non-cancer tissues were obtained from 33 patients and were used to construct tissue microarrays. The expression levels of these markers in the tissue microarrays were measured by enzyme-linked Immunohistochemistry assay, and the relationships were analyzed by Pearson’s chi-square test and two-tailed t-test analysis.

**Results:**

We demonstrated for the first time that TNS2 is a phosphorylation substrate of Axl. Moreover, we found a positive relationship between TNS2 expression and the expression of Axl, IRS-1, PDK1 and Glut4 in pancreatic cancer patients. Based on these results, we suggest that Axl modulates glucose metabolism potentially through TNS2 and IRS-1. We hypothesize that there exists a novel mechanism whereby Axl binds to and phosphorylates TNS2, releasing TNS2 from interaction with IRS-1 and resulting in increased stability of IRS-1. The two key enzymes of aerobic glycolysis (Glut4 and PDK1) were found to be up-regulated by Axl/TNS2/IRS-1 cross-talk and may play a critical role in glucose metabolism of cancer cells.

**Conclusions:**

Our results revealed for the first time that Axl binds to and phosphorylates TNS2 and that Axl/TNS2/IRS-1 cross-talk may potentially play a critical role in glucose metabolism of cancer cells.

**Electronic supplementary material:**

The online version of this article (10.1186/s12929-018-0465-x) contains supplementary material, which is available to authorized users.

## Background

Axl is a receptor tyrosine kinase, which is characterized as a pro-oncogenesis factor involved in cancer progression. Along with its ligand Gas6, Axl, through its signaling, plays important physiological roles in the nervous [1], reproductive, and vascular systems [2]. Axl is also known to be associated with hemostasis, inflammation, autoimmune diseases [1–3] and pancreatic cancer [4, 5]. At high-glucose conditions, Axl binds to and conveys signals through phosphatidylinositol-3-kinase (PI3K), resulting in apoptosis of vascular smooth muscle cells. At low-glucose conditions, Axl binds to the protein tyrosine kinase SHP2;this results in increased cell migration (diabetic cardiovascular disease) and survival (anti-apoptosis) [2]. However, whether Axl is involved in cancer metabolism for cancer progression remains largely unknown. In this study, for the first time, we demonstrate that Axl may affect glucose metabolism through TNS2.

Tensin2 — also known as TNS2, TENC1, and C1-TEN — is a member of the Tensin family that also includes tensin1, tensin3 and tensin4/CTEN. All four members can be found in focal adhesions and fibrillar adhesions, and they can modulate both cell motility and transformation [6]. TNS2 contains a Src Homology 2 (SH2) domain and a phosphotyrosine-binding (PTB) domain, both of which allow it to interact with tyrosine-phosphorylated proteins such as the PI3K/Akt kinase, p130Cas, and focal adhesion kinase (FAK) [6, 7]. It is unclear whether TNS2 is regulated by these tyrosine-phosphorylated proteins. TNS2 has been identified as a binding partner for several different kinases and proteins, including Axl [8, 9], the non-receptor tyrosine kinase Syk [10], thrombopoietin receptor Mpl [11], insulin receptor substrate 1 (IRS-1) [12], deleted in liver cancer 1 (DLC1) [13–15], and disrupted in schizophrenia 1 (DISC1) [16]. It is plausible that TNS2 conveys different signals by binding with different partners involved in cancer progression. For instance, the tumor suppressor DLC1 may interact with TNS2 and contribute to the growth-suppressive activity of DLC1 in hepatocellular carcinoma [13]. Silencing TNS2 expression may increase the activities of Akt, Mek, and IRS-1 in lung and cervical cancers [17]. However, Jung et al. [11] demonstrated that another consequence of TNS2 binding to Mpl is recruitment of PI3K to the plasma membrane, where it can phosphorylate PIP2, thereby activating the downstream effector AKT/PKB in platelet disorders. The role of TNS2 in cancer remains a mystery. TNS2 interacts not only with different binding partners but also with different isoforms. TNS2 has three different isoforms, variant 1 to 3 (V1, V2, and V3). High expression of TNS2 V1 and V3 has been found in skeletal muscle, whereas TNS2 V2 has been found in pancreas and heart [17]. In this study, we examined the signaling effects of the TNS2 V2 isoform in pancreatic cancer cell lines.

Although a previous study found that TNS2 may bind with Axl in yeast two-hybrid interactions [8], the details of the interaction remain to be elucidated. In our research, we demonstrate that TNS2 serves as a substrate for Axl and may play a critical role in cancer by modulating Axl signaling. The pancreas secretes insulin and glucagon, two of the hormones that regulate glucose metabolism. Notably, pancreatic tumors heavily rely on glycolysis [18]. Therefore, we further examined the correlation of TNS2 expression and expression of Axl, IRS-1, pyruvate dehydrogenase kinase 1 (PDK1) and glucose transporter type 4 (Glut4) in tissue of 33 patients with pancreatic cancer. PDK1 is a critical determinant of glucose metabolism and Warburg-like metabolic changes (or aerobic glycolysis) [19, 20]. Our results suggest that Axl binds to and phosphorylates TNS2 and is involved in glucose metabolism in human pancreatic cancer cells.

## Methods

### Cell culture and treatment

HEK293T, human osteosarcoma U2OS cells and human pancreatic cnacer Panc-1 cells were grown in DMEM supplemented with 10% fetal bovine serum (FBS) and 1% penicillin/streptomycin (Invitrogen, USA). Mia PaCa-2 cells were cultured in DMEM supplemented with 2.5% horse serum, 10% FBS and 1% (*w*/*v*) penicillin/streptomycin (Invitrogen, USA). Cells were routinely grown in a humidified incubator with 5% CO_2_ at 37 °C. Following transfection with various plasmids for 24 h, cells were treated with/without 400 ng/mL recombinant human Gas6 (R&D Systems Inc., Minneapolis, MN) for 15 min. The cells were then harvested and analyzed for gene expression by Western blot. All these cell lines were authenticated by Mission Biotech (Taipei, Taiwan) and DNA typing was analyzed with ABI PRISM 3730 Genetic Analyzer and GeneMapper software V3.7.

### Plasmid constructs

The Axl recombinant plasmid and shRNA expression vecter constracts were followed in our previous publish [21]. Wild-type human Tensin2 plasmid (KIAA1075), which was cloned into pCMV-Myc mammalian expression vector, was a gift from Drs. B. Dahlbäck and S. Hafizi. A series of mutation variants were cloned into the pCDNA3.0 vector (Novagen, Australia). The site-directed mutagenesis for the generation of Myc-TNS2 (C231S) and Myc-TNS2 (Y483S) was performed with a KOH Hotstar DNA polymerase kit (Merck Millipore, Darmstadt, Germany) using the following primers:

- 5′- GGAT GGC AGTCCTTTTGCCCAGGTGCAGC G-3’ and
- 5’-CGCTGCACCTGGGCAAAAGGACTGCCATCC-3’ (for Y483F); and
- 5’-GTGGTCGTACTATACAGCAAGGGAAACAAGGGC-3’ and
- 5’-GCCCTTGTTTCCCTTGCTGTATAGTACGACCAC-3’ (for C231S)

### Immunoprecipitation

Cells were transfected as aforementioned and grown in 10-cm dishes. To extract proteins, cells were washed with ice-cold PBS and lysed in NETN lysis buffer (150 mM NaCl, 1 mM EDTA, 20 mM Tris-Cl pH 8.0, 0.5%NP-40(*v*/v) containing protease and phosphatase inhibitors (50 mM NaF, 0.1 mM Na_3_VO4). The lysates were incubated, respectively, with 2 μg of IP antibodies to Myc (9E10) (#05419, Millipore) or Axl (C-20) (#sc-1096, Santa Cruz) at 4 °C overnight with constant rotation. The immunocomplex was captured by incubating with 40 μl protein A/G-agarose beads (Sigma Aldrich, Sr. Louis, MO, USA) for 3 h at 4 °C with constant rotation. Following 1 min centrifugation at 10000 g, the beads were washed three times with ice-cold NETN buffer and ready for SDS-PAGE analysis or kinase assay. Representative Western blot results are shown in figures and experiments were performed in triplicate.

### Immunoblotting

Cell extracts were subjected to Western blot analysis as previously described [21]. The monoclonal antibody against the Myc-epitope (9B10) was from Millipore Technology. The expression vector of TNS2 was Myc-tagged and cloned into the pcDNA3.0 plasmid. To generate an antibody against p-Axl, the synthetic phospho-oligopeptide Asp-Gly-Leu-(phospho)Tyr-Ala-Leu-Met-Ser-Arg-Cys was used as an antigen for the generation of rabbit immune serum using a commercial service (GenTex Inc., California). The following antibodies were used: Axl (C-20), Erk sc-94, Santa Cruz), p-Erk (sc-7383, Santa Cruz), Akt 1/2/3 (#9272, Cell signaling) and p-Akt (#4051, Cell signaling), IRS-1 (#06–248,Millipore,), IRS-2 (#06–506, Upstate), TNS2 (SAB4200268, Sigma Aldrich), pTNS2-Y483 (ab138414, Abcam, Eugene), PDK1 (#21005, SAB signalway) and Glut4 (#2231S, Cell signaling).

### In vitro kinase assay

The active His-tagged human recombinant Axl kinase (473-end) was produced from insect Sf9 cells and purchased from SignalChem Life Sciences (Richmond, CA). HEK293T cells were transfected with Tensin2 expression vector and immunoprecipitated for 24 h. The active Axl kinase and Tensin2 proteins (as substrates) immunoprecipitated from the transfected HEK293T cells were incubated with 10 μCi [γ-32^P^] ATP, phosphatase inhibitors (10 mM NaF, 50 mM β-glycerophosphate) in kinase buffer (25 mM HEPES pH 7.5, 50 mM NaCl, 10 mM MgCl_2_, 1 mM DTT, 10 μM ATP) at 30 °C for 40 min, followed by adding SDS-PAGE sample buffer to stop the reaction. Phosphorylated radioactive proteins were separated by SDS-PAGE and detected by autoradiography. R428 is a specific Axl inhibitor. Representative results are shown in Fig. 3c and experiments were performed in triplicate.

### Glucose uptake assay

For the basal glucose uptake assay, U2OS and Mia PaCa-2 cells were seeded in 6-well plates (5 × 10^5^ cells per well) and cultured for 24 h, washed with phosphate-buffered saline (PBS) and incubated for 3 h with serum-free medium. All cells were transfected with pcDNA3.0 vector (vehicle) or Tensin2 expression plasmid with or without Axl expression plasmid for 24 h and then washed with PBS and incubated for 3 h with serum-free medium. Glucose uptake was measured using a glucose uptake cell-based assay kit (Cayman Chemical, MI) according to the manufacturer’s instructions. Three replicates were performed for each group and the experiments were repeated three times to confirm the results. The BD Bioscience FACSCalibur flow cytometry was used for glucose uptake analysis.

### Colony formation

Mia PaCa-2 cells were seeded in a 6-well dish (100 cells per well) overnight, then transfected with various expression vectors as indicated and allowed to grow for 11 d in a humidified incubator with 5% CO_2_ at 37 °C. Representative colony results are shown in figures and experiments were performed in replicate.

### Patients and tissue microarrays

The tissue microarrays were constructed using formalin-fixed, paraffin-embedded archival tissue blocks from 33 patients who had undergone pancreaticoduodenectomy at Cardinal Tien Hospital between 2006 and 2013. We excluded patients who had received chemotherapy or radiation therapy before resection. All patient clinical charts and histopathology reports were reviewed for tumor size, extracapsular invasion, lymph node metastasis or distant metastasis, and TMN stage. For each patient, 2 cores of tumor and 2 cores of paired non-neoplastic pancreatic tissue were sampled from representative areas using a 2.0-mm punch. Due to the heterogeneity of the immunohistochemical staining patterns that are associated with adenocarcinoma, we determined tissue microarray slides via standard hematoxylin and eosin (H&E) staining and inserted the samples into recipient paraffin blocks to form complete tissue arrays. For each sample selected on an array slide, the carcinoma type, degree of cell differentiation, growth pattern, tumor cell nuclear morphology, degree of metaplasia, degree of calcification, extent of necrosis, mitosis count, and other specific indices of differentiation were rechecked by two pathologists. Sections of 4 μm were cut from these complete array blocks and transferred to salinized glass slides. All procedures involving patient tissues were delinked anonymously for protection and approved by the Institutional Review Board (IRB) of Cardinal Tien Hospital (CTH-101-3-5-054).

### Immunohistochemical analysis for gene expression

IHC staining was performed using a Ventana BenchMark XT automated stainer (Ventana, Tucson, AZ). The sections were incubated respectively with various antibodies against Akt 1/2/3, p-Akt 1/2/3, Erk, p-Erk, IRS-1, PDK1, PKM2, and TNS2 for 1 h at room temperature. For Axl, p-Axl and p-TNS2 detection, the sections were incubated overnight at 4 °C with anti-AXL (1:50, ab72069, Abcam, Eugene, OR), anti-pAxl-Y779 (1:100 AF2228, R&D, MN, USA) and anti-p-TNS2 (1:100, ab138414, Abcam, Eugene, OR), respectively. Antibody binding was revealed using the Strepta ABComplex/HRP Duet kit (Dako, Santa Barbara, CA, USA). To evaluate Axl and other protein expression in pancreatic ductal adenocarcinoma (PDAC) cells and non-neoplastic pancreatic epithelial cells, the IHC staining was rechecked by two pathologists. The immunostaining results were scored quantitatively based on staining intensity and the percentage of positively stained cells. An H-score ranging from 0 to 300 was calculated by multiplying the staining intensity score (0 for no staining, 1 for faint staining, 2 for moderate staining, and 3 for strong staining) by the percentage of positively stained cells (ranging from 0 to 100). PDAC cases were categorized into 3 groups based on H-score: low expression (H-score 0—100), moderate expression (H-score 101—200), and high expression (H-score 201—300). The immunohistochemically stained slides of PDAC tissue microarrays were scanned at 200× magnification with a Pannoramic MIDI II digital slide scanner (3DHISTECH, Budapest, Hungary).

### Statistical analysis

The densitometric quantification of Western blot was performed by Image J and analyzed by t-test. Gene expression in PDAC and adjacent non-tumor tissues was analyzed by Pearson’s chi-square statistics. The correlation between different genes in PDAC cancer samples were analyzed by two-tailed t-test analysis. All differences were considered statistically significant when *p*-value < 0.05.

## Results

### Expression of TNS2 and Axl in pancreatic cancer

**Fig. 1:**
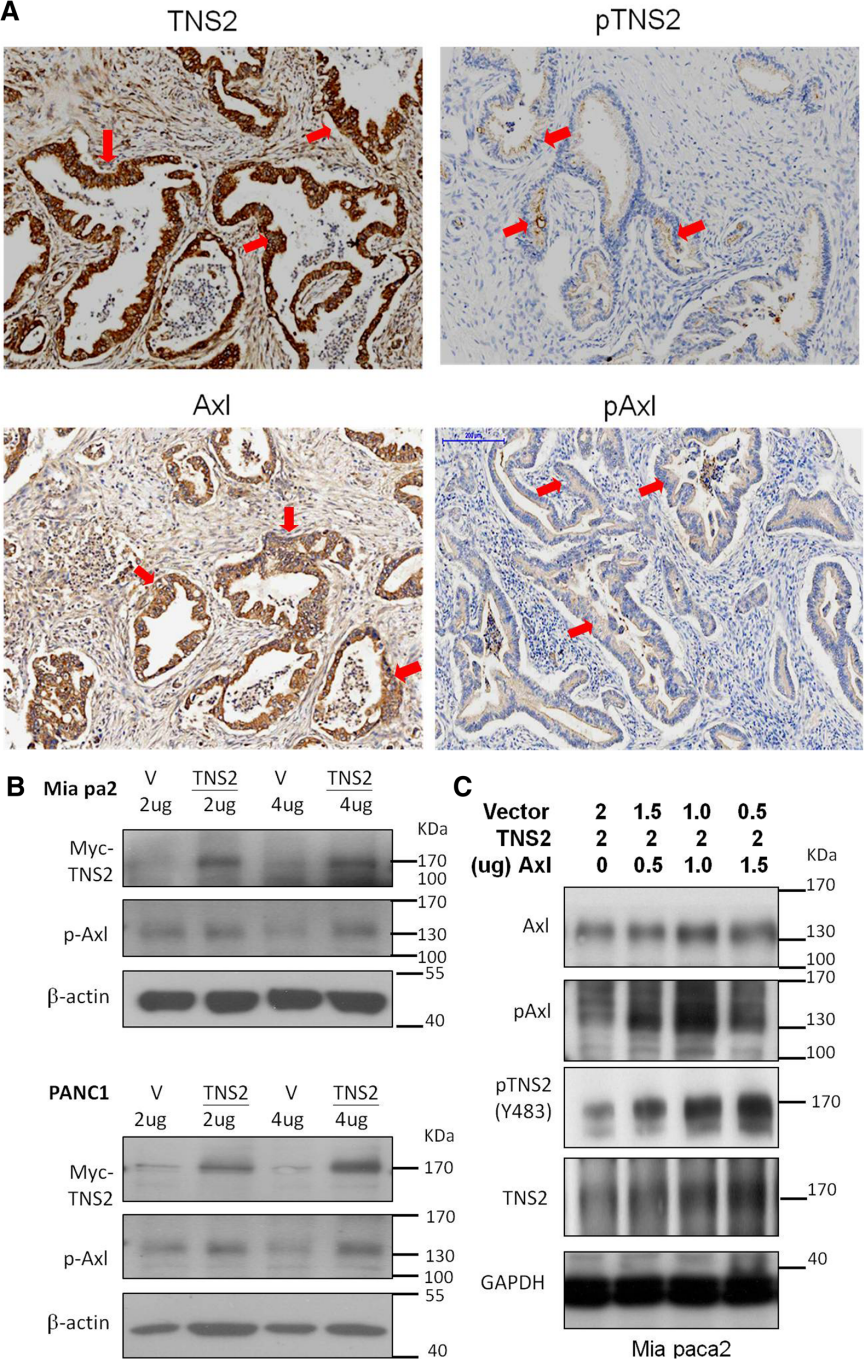
Expression of TNS2 and Axl in pancreatic cancer. **a** Representative micrographs show the histopathologic feature and the expression of TNS2 and AXL in 33 PDAC tumor tissues by IHC. Scale bar, 200 μm (**b**) Mia PaCa2 and PANC1 pancreatic cancer cells were transfected with the Myc-tagged wild type TNS2 expression plasmid or the empty vector control pcDNA3 and analyzed by Western blot 24 h later. **c** Mia PaCa2 cells were transfected with Axl or TNS2 or both followed by Western blot analysis 24 h later

TNS2 has been demonstrated to bind with Axl [8] and down regulates Akt expression [22]. The TNS2 expression status of five pancreatic cancer cell lines was determined by Western blot analysis. While AsPC1, HPDE, and SUIT-2 expressed little TNS2, the two relatively drug-resistant cell lines, Mia PaCa-2 and PANC1, were found to express higher levels of TNS2 (Additional file 1). In the tumor tissue microarray, high expression levels (H-score 201—300) of TNS2, p-TNS2, Axl, and p-Axl were detected in the pancreatic ductal areas of 33 PDAC patients’ tumor tissues (Fig. 1a). Ectopic expression of TNS2 did not significantly alter the levels of p-Axl (Fig. 1b). When Axl and the Myc-tagged TNS2 expression constructs were co-transfected into Mia PaCa-2 cells, the levels of both TNS2 and p-TNS2 were significantly increased by Axl transfection in a dose-dependent manner (0 to 1.5 μg of Axl plasmid DNA) (Fig. 1c).

### Axl knockdown abrogates TNS2 expression and down-regulates its downstream signaling and cellular glucose import

**Fig. 2:**
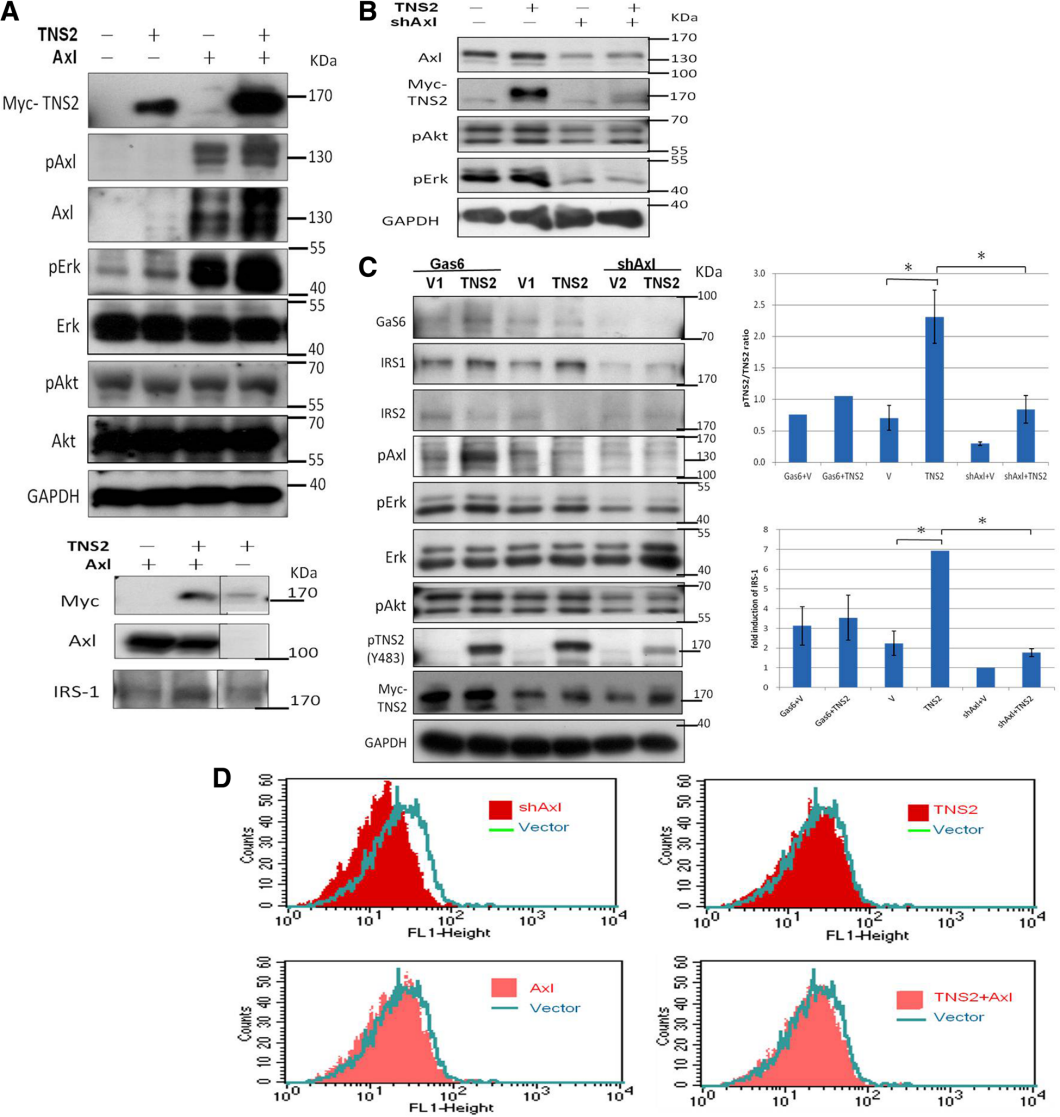
TNS2 interacts with Axl and up-regulates its downstream signaling and cellular glucose import. Effects of TNS2 and Axl expression on downstream signaling. **a** HEK293T cells were transfected with 2 μg Axl or 2 μg TNS2 or both (1 μg Axl with 1 μg TNS2) followed by Western blot analysis 24 h later. **b**, **c** Western blot analyses were performed in U2OS cells 24 h after transfection with TNS2 expression plasmid with or without shAxl plasmid. Recombinant human Gas6 were treated at 400 ng/mL for 15 min where indicated. Representative Western blot results are shown here and experiments were performed in triplicate. Quantitative data of the pTNS2/TNS2 ratios and the IRS-1 expression were presented in bar graphs. Data were obtained from three independent experiments and were presented as mean ± SEM. *p < 0.05. **d** After transfection with Axl or shAxl or vector control, U2OS cells were pre-incubated in growth medium without glucose for 1 h and then treated with 2-NDBG for 10 min followed by flow cytometry analysis

When Myc-TNS2 and Axl were co-transfected into the non-Axl-expressing HEK293T cells, p-Axl expression was markedly higher in co-transfected cells compared with that in cells with Myc-TNS2 alone or Axl alone (Fig. 2a). Similar results were observed for the levels of p-Erk but not for the levels of p-Akt, both of which are Axl downstream molecules. (Fig. 2a). To further confirm that Axl indeed positively regulates TNS2, we knocked down Axl expression in Axl-context cell, U2OS cell line, and also examined TNS2 and glucose import-related molecules by Western blot analysis. Expression of TNS2 was drastically down-regulated by shAxl co-transfection (Fig. 2b, lanes 2 and 4). Similarly, levels of p-Erk were downregulated by shAxl co-transfection. Because the transfected AXL and TNS2 contain neither their own promoters nor their own mRNA UTRs, the transfection effect apparently results from a post-translational mechanism (i.e., at the protein level). To distinguish ligand-induced Axl phosphorylation and ligand-independent Axl autophosphorylation, we utilized Gas6, the Axl ligand, to activate the Axl signaling pathway. Unfortunately, Gas6-induced Axl is not markedly upregulated in IRS1, p-Akt, or p-Erk after Gas6 treatment (despite Axl activation) (Fig. 2c) compared with Fig. 2a. We speculate that the effect observed was probably mediated through a ligand-independent pathway. Overall these results suggest that Axl positively regulates TNS2 expression. Since both Axl and TNS2 have been reported to be involved in glucose metabolism [2, 23], we also assessed IRS-1 expression and glucose uptake. The fold inductions of IRS-1 increased significantly upon TNS2 overexpression in cells (Fig. 2a lower panel, Additional file 2, and Fig. 2c). Consistently, knockdown of Axl expression abrogated TNS2 expression and significantly decreased cellular glucose import (Fig. 2d, upper left panel). Overexpression of Axl and/or TNS2 did not further enhance glucose uptake, likely because the endogenous levels of Axl and TNS2 expression were already sufficient for U2OS cells in terms of glucose uptake (Fig. 2d, lower panels).

### Axl directly phosphorylates TNS2 and mutation of TNS2 at Y483 attenuates cancer cell proliferation

**Fig. 3:**
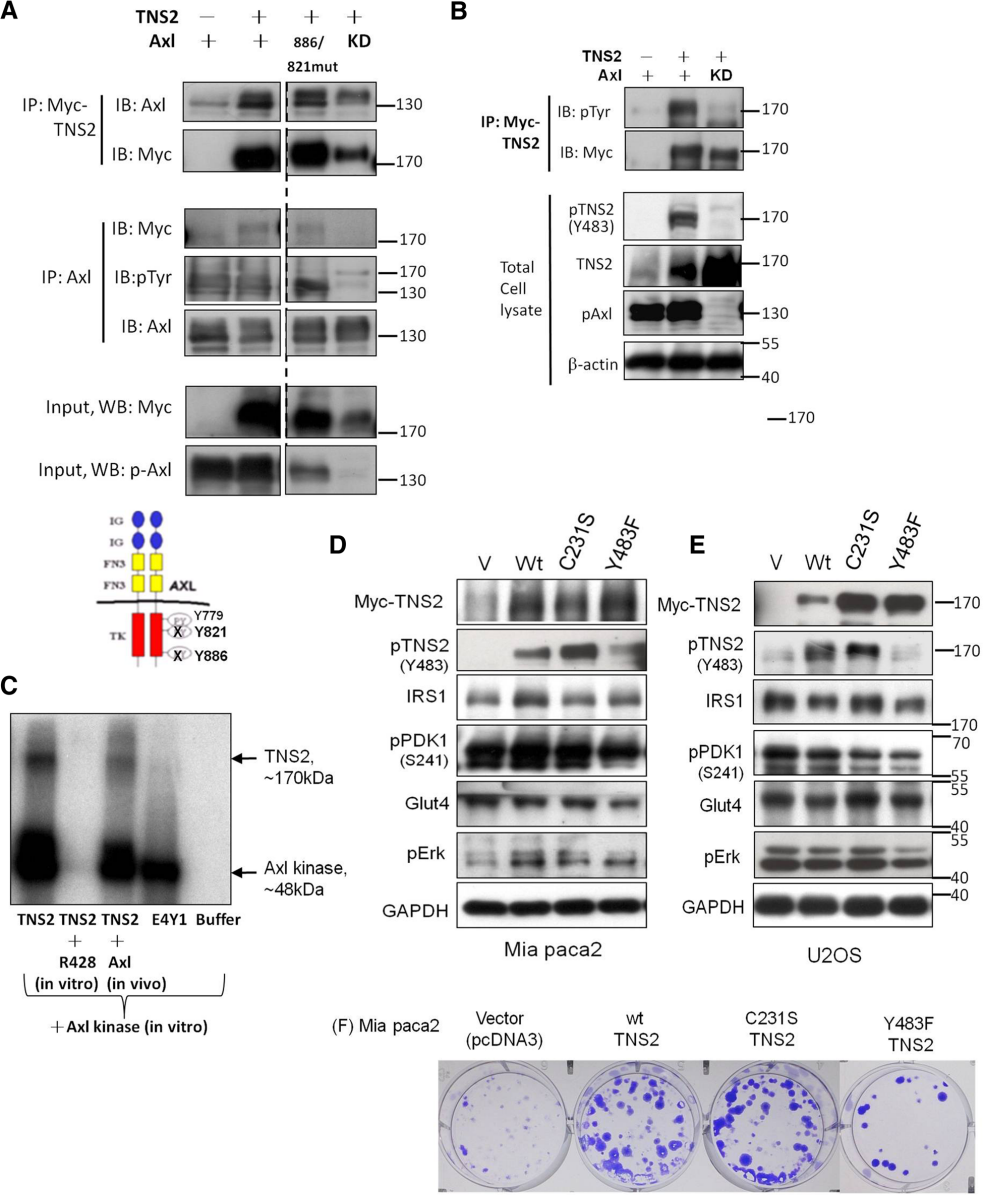
Axl binds to and phosphorylates TNS2 at tyrosine 483 and regulates cancer cells proliferation*.* **a** To demonstrate the binding of TNS2 to Axl, HEK293T cells were transfected with TNS2 or Axl and lysed. TNS2 was co-immunoprecipitated (co-IP) by Myc and Axl. **b** To determine the effect of phosphorylation of Axl in activation of TNS2. HEK293T cells were transfected with TNS2 or Axl and lysed. TNS2 was IP by Myc, and blotted sequentially for phosphotyrosine (pTyr) and Myc. Total cell lysates were subjected to immunoblotting with related gene expression. **c** TNS2 is a substrate for Axl. TNS2 protein was pulled down from U2OS cells 24 h after being transfected with TNS2, followed by in vitro kinase assay. Poly(E4Y1) is an exogenous substrate for positive control. R428 is a specific Axl inhibitor. Effects of wild-type and the phosphatase-dead C231S and the phosphorylation mutant Y483F of TNS2 on the expression of various markers in Mia PaCa2 (**d**) and U2OS cells (**e**). All cells were transfected with various plasmids, respectively, and subjected to Western blot analysis 24 h later. **f** Effects of wild-type and the phosphatase-dead TNS2 (C231S) and the phosphorylation-dead TNS2 (Y483F) on colony formation in Mia PaCa2 cells. KD, kinase-death; Wt, wild type

Reciprocal co-IP analysis was first performed to confirm the interaction between Axl and TNS2 in HEK293T cells (Fig. 3a and -Additional file 2). Axl has three auto-phosphorylation sites: Y779, Y821, and Y886. Y779 is important in Axl kinase activity [24]. We verified whether Y779 regulates phosphorylation of TNS2. The result demonstrated that all three sites are required for TNS2 phosphorylation and not just Y779. The Y886F/Y821F double mutation did not affect the interaction between Axl and TNS2, whereas the kinase-death (KD) mutation (K567R) significantly abolished the interaction (Fig. 3a). Most importantly, we observed that Axl enhances TNS2 phosphorylation when the IP assay was conducted with p-tyrosine (Fig. 3b, upper panel) and western blot with anti-p-TNS2 (Fig. 3b, lower panel). We further demonstrated that Axl directly phosphorylates TNS2 by an in vitro kinase assay. The cell was transfected with Myc-TNS2 and then pulled down with Myc. The Myc-TNS2 was phosphorylated by the addition of Axl kinase in vitro*,* but its phosphorylation was completely abolished by the specific Axl inhibitor R428 (Fig. 3c, lanes 1 and 2). This result suggested that TNS2 is a substrate of Axl kinase. Moreover, it only slightly bound when we co-transfected Axl and TNS2 in vivo in the culture dish for the same assay.

According our data, the dysregulation of the Axl kinase is involved in phosphorylation of TNS2. To determine the certain phosphorylation site of TNS2, we constructed two candidate mutations of TNS2, TNS2-Cysteine 231 (C231S) and TNS2-tyrosine 483 (Y483F). In previous studies, TNS2(C231), a secondary P-loop Cys, has been shown to be crucial for the phosphatase activity of TNS2 [12, 25]. It has been reported that TNS2 (Y483) is a phosphorylation site for Src [26, 27]. According to the previous study, C231S is a phosphatase-dead mutant of TNS2 and Y483F is a phosphorylation-dead mutant of TNS2. Here, we assessed the potential involvement of several insulin-signaling molecules in both Mia PaCa-2 and U2OS cells, which express low levels of endogenous Axl. Phosphorylation of TNS2 was abolished in the Y483F mutant but not in the C231S mutant (Fig. 3d and e). We further measured the IRS1-associated signaling during dysregulation of TNS2. Expressions of glucose metabolism-related proteins, IRS-1, Glut4, and PDK1, were decreased in Mia PaCa-2 and U2OS cells expressing TNS2 (Y483F) compared with that in cells expressing TNS2 (wild type) (Fig. 3d and e). More importantly, the mutant Y483 site of TNS2 reflected less colony formation (Fig. 3f). The trend of colony-formation ability was consistent with the expression levels of IRS-1, Glut4, and PDK1 (Fig. 3d and f). Based on these findings, the expression level of TNS2 and its associated, IRS-1, Glut4 and PDK1 were found to be crucial for colony formation.

### Clinical association of TNS2 and Axl expression in human pancreatic ductal adenocarcinomas

**Fig. 4:**
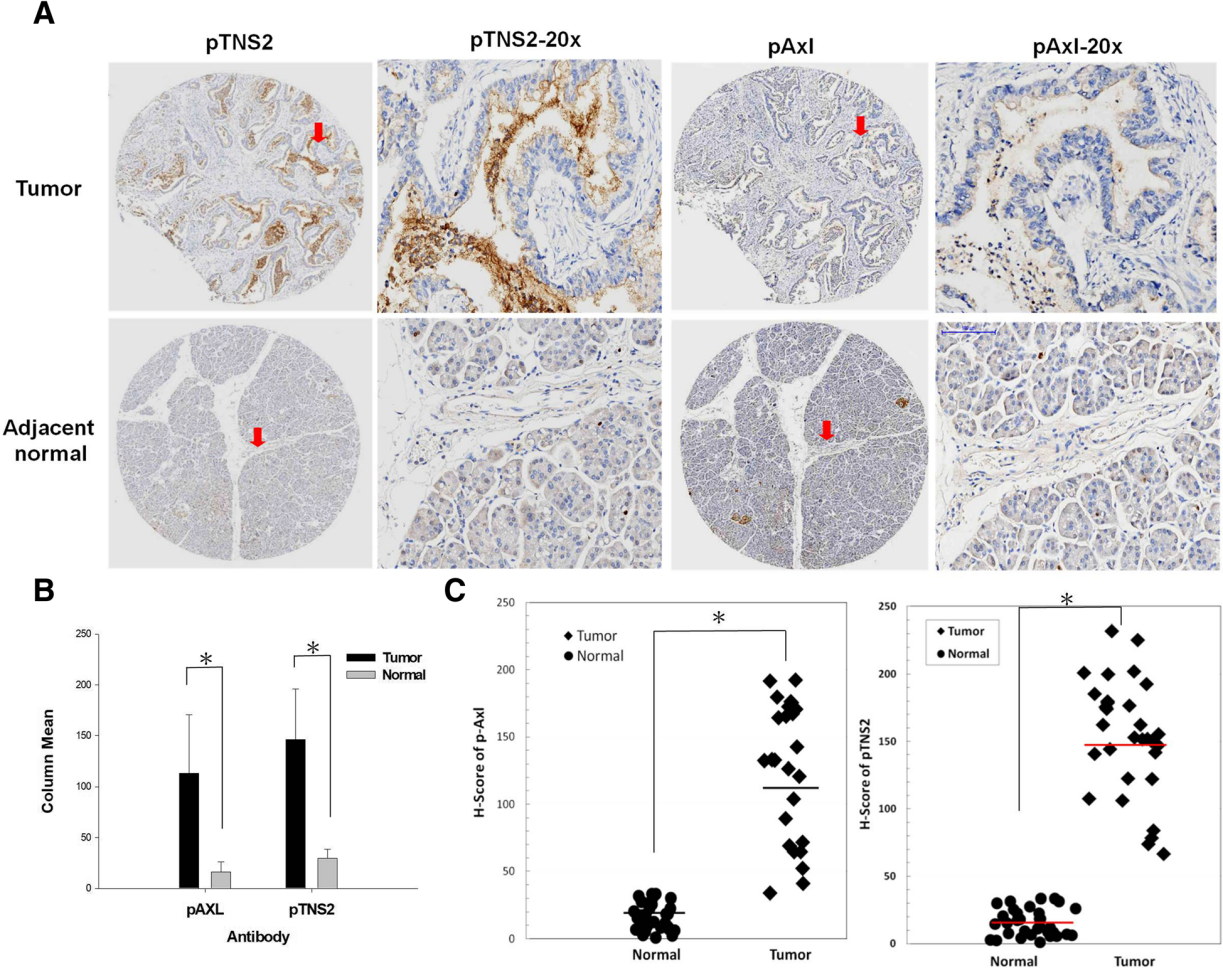
Analysis of p-TNS2 and p-Axl expression in tumor and adjacent non-tumor tissues of the human pancreatic ductal adenocarcinomas. Both p-Axl and p-TNS2 proteins were upregulated by IHC in 33 PDAC. **a** Representative micrographs show the histopathologic features and the expressions of p-TNS2 and pAxl in adjacent normal and tumor tissues by immunohistochemical analysis. For each section’s micrograph, a higher magnification is shown to its right (indicated by an arrow) for clarity. The adjacent normal tissue showed low protein expression in pancreatic ductal epithelial cells but high expression in endocrine acinar cell. Scale bars, 100 μm. **b** The bar chart shows Mean ± SD. **c** The dots chart shows the H-score of individual patients and the median values of groups (*p* < 0.05)

We performed tissue microarray immunohistochemical analysis on 33 paired human PDAC samples and adjacent non-tumor tissues. The clinicopathological features of the 33 patients were stratified into four stages: 30.3% (11/33) stage I, 60.6% (20/33) stage II, 0% (0/33) stage III, and 6% (2/33) stage IV. There were no statistically significant differences (among all four groups, *p* > 0.05) between early (IIa and earlier) and late stages (IIb and later) in the expression of Axl, TNS2, IRS1, and Glut4 proteins (Additional file 3). Consistent with the Western blot data, p-TNS2 and p-Axl were highly correlated (*p* < 0.0005, Table 1). In the adjacent normal pancreatic tissues, p-TNS2 and p-Axl were highly expressed in the endocrine acinar cells but had low expression in the ductal epithelial cells. [4, 5] Representative micrographs of TNS2, Axl, and p-TNS2, p-Axl are shown in Figs. 1a and 4a. Quantitative analysis showed that the IHC-score of p-Axl and p-TNS2 in patient tumors was significantly greater than those of patient adjacent normal tissues (Fig. 4b and c). Consistent with TNS2 and Axl expression, the insulin signaling-related molecules, including — IRS-1 and Glut4 — had a similar trend in ductal areas (Additional file 4). Furthermore, statistical analysis of PDAC patients’ tissue microarray IHC scoring showed that TNS2 was positively associated with the glucose metabolism-related IRS-1 and Glut4, the proliferation marker ki-67, the angiogenesis marker CD31, and the mesenchymal markers N-cadherin and fibronectin (Table 2, left); in addition, Axl was positively associated with the mesenchymal marker fibronectin (Table 2, right). Similar analysis showed that p-TNS2 was positively associated with the cytoplasmic p-Akt, and that p-Axl was positively associated with N-cadherin, p-Akt and IRS-1 (Table 3). These results suggested that the expression of TNS2 may involved in pancreatic cnacer metabolism, proliferation, angiogenesis, and epithelial–mesenchymal transition (EMT) process.

**Table 1:** Correlation between Axl, p-Axl, TNS2, and p-TNS2, as examined using pancreatic tissue array slides

	*r*	*p*
*Tensin2*		
Axl	0.198	0.270
pAxl (Y779)	0.199	0.268
pTensin2 (Y483)	- 0.130	0.471
*pTensin2*		
Axl	0.069	0.702
pAxl (Y779)	0.629	0.000*
Tensin2 (Y483)	- 0.130	0.471

The correlations were evaluated using the Pearson correlation test. Patient number = 33, *p > 0.0005

**Table 2:** Correlation between Axl or TNS2, and various markers using pancreatic tissue arrays

	*r*	*p*
*Tensin2*		
Glut4	0.537	0.001*
IRS-1	0.335	0.043*
Ki-67	0.432	0.012*
CD31	0.435	0.011*
E-cadherin	0.289	0.102
N-cadherin	0.452	0.008*
Fibronectin	- 0.361	0.039*
Caspase3	- 0.083	0.644
pAkt (nuclei)	0.297	0.093
pAkt (cytoplasm)	- 0.029	0.872
pErk (nuclei)	0.182	0.319
pErk (cytoplasm)	0.229	0.200
*Axl*		
Glut4	0.247	0.165
IRS-1	0.133	0.461
Ki-67	0.119	0.509
CD31	0.251	0.159
E-cadherin	0.072	0.690
N-cadherin	- 0.168	0.350
Fibronectin	- 0.345	0.049*
Caspase3	0.149	0.408
pAkt (nuclei)	0.058	0.746
pAkt (cytoplasm)	0.217	0.224
pErk (nuclei)	0.186	0.307
pErk (cytoplasm)	0.033	0.856

The correlations were evaluated using the Pearson correlation test

Glut4 and IRS1 are involved in glycolysis; Ki-67, proliferation marker; CD31, angiogenesis marker; E-cadherin, epithelial marker; N-cadherin and fibronectin, mesenchymal marker; Caspase 3, apoptosis marker. Patient number = 33, *p < 0.05

**Table 3:** Correlation between p-Axl or p-TNS2 and various markers using pancreatic tissue arrays

	*r*	*p*
*pTensin2*		
Glut4	- 0.037	0.082
IRS-1	0.003	0.988
Ki-67	0.106	0.556
CD31	- 0.073	0.683
E-cadherin	0.079	0.661
N-cadherin	0.082	0.649
Fibronectin	0.179	0.321
Caspase3	0.263	0.139
pAkt (nuclei)	- 0.039	0.830
pAkt (cytoplasm)	0.521	0.002*
pErk (nuclei)	0.286	0.112
pErk (cytoplasm)	0.018	0.919
*pAxl*		
Glut4	- 0.058	0.750
IRS-1	0.273	0.125
Ki-67	0.138	0.443
CD31	0.209	0.244
E-cadherin	0.037	0.836
N-cadherin	0.437	0.011*
Fibronectin	0.028	0.875
Caspase3	0.087	0.631
pAkt (nuclei)	0.423	0.014*
pAkt (cytoplasm)	0.749	0.000*
pErk (nuclei)	0.211	0.246
pErk (cytoplasm)	0.286	0.107

The correlations were evaluated using the Pearson correlation test

Glut4 and IRS1 are involved in glycolysis; Ki-67, proliferation marker; CD31, angiogenesis marker; E-cadherin, epithelial marker; N-cadherin and fibronectin are mesenchymal markers; Caspase 3, apoptosis marker. Patient number = 33, *p < 0.05

## Discussion

The receptor tyrosine kinase Axl is a well-known molecule that promotes tumor progression in various cancers. Cancer cells rewire their metabolism to promote cancer progression; one of the common features of this altered metabolism is increased glucose uptake and the conversion of glucose to lactate [28]. However, the function of Axl in cancer metabolism is not evident. Based on previous reports, surviving dormant cancer cells may over-express insulin-like growth factor-1 receptor (IGF-1R) during recurrence [29] and Axl may mediate different signals in high and low glucose situations [2, 30]. The results of our present study suggest that knockdown of Axl expression reduces glucose uptake and that Axl is related to high expression of glucose metabolism molecules, IRS-1, Glut4 and PDK1 in cancer tissues. We investigated the possible mechanism of Axl-regulated cancer metabolism through TNS2 expression. Additionally, we identified TNS2 as a substrate of Axl (Fig. 3c). TNS2 region contains both an SH2 domain and a PTB domain, suggesting that an interaction with Axl might be mediated by phosphotyrosine. Axl enhanced TNS2 expression in Fig. 2a and kinase-dead of Axl abrogates TNS2 expression in Fig. 3b. According to these results, we suggested that Axl may regulate the stability of TNS2 through the interaction and phosphorylation of TNS2. The mechanism might involved in regulation of TNS2 degradation and stability of TNS2 expression. Unexpectedly, the results of the downstream molecules of Axl, p-Akt and p-Erk [31–33], were conflicting from cell culture and tissue specimens. Both p-TNS2 and p-Axl were positively associated with the cytoplasmic p-Akt but not p-Erk (Tables 2 and 3). However, there is no effect of overexpression of myc-TNS2 and Axl on p-Akt levels (Fig. 2a and c). The results of studies involving human embryonic kidney 293 T (Fig. 2a), human osteosarcoma U2OS, and human pancreatic cancer specimens (Tables 2 and 3) are conflicting. Although Ara Koh et al. reported that IRS-1 regulates p-Akt in type 2 diabetes mellitus [12], its possible mechanism in cancer is yet to be elucidated. In addition, Cavet et al. reported that at high-glucose condition, Axl modulates p-Erk to vascular smooth muscle cell migration, whereas at low-glucose condition, Axl modulates p-Akt [2]. Both p-Akt and p-Erk are modulated among various tissues as well as different glucose conditions. In our data, we cultured all cells in high-glucose DMEM medium. However, we did not investigate the glucose condition and/or hypoxia conditons of the patients suffering from pancreatic cancer. We speculate that these results may related to the cell contexts of the cell lines and the high-glucose culture conditions we used.

IRS-1 is a critical mediator in insulin signaling that regulates glucose uptake and PDK1-mediated glucose metabolism [34]. A recent report indicated a novel mechanism which TNS2 mediates IRS-1 degradation via dephosphorylation at IRS-1 Y612. This mechanism is different from the canonical insulin/IGF-1-induced IRS-1 degradation via phosphorylation at certain serine sites of IRS-1 in diabetes patients [12]. Several proteins have been found to interact with TNS2 through its C231 site of the PTPase domain [8, 25]; this C231 mutation may abolish the tumorigenicity of breast and colon cancer cells [17]. The Oncomine reference database was used to explore the effects of TNS2 in cancer cells. Its role in various cancer types is not definitively established yet; moreover, TNS2’s role in PDAC remains unknown (Additional file 5). In this study, however, we demonstrate for the first time that Axl directly binds to and phosphorylates TNS2 at Y483. The Y483 site is close to the PTPase domain of TNS2. The PTPase domain is critical for the de-phosphorylation of certain tyrosine kinases and for the tumor-suppressive functions of TNS2. The role of TNS2-Y483 in the insulin signaling pathway is not yet fully characterized. Our results show that the Y483 site is more important than the C231 site of TNS2 for regulation of IRS-1 expression and IRS-1-mediated signaling. By using tissue microarrays of pancreatic patients, we also demonstrate that in comparison to the adjacent normal parts of pancreaatic patients, cancer cells highly express both Axl (total form and the Y799-phosphorylated form) and TNS2 (total form and the Y483-phosphorylated form), as well as other molecules involved in glucose metabolism and cancer progression. In the future, it will be interesting to investigate whether Y483 phosphorylation of TNS2 plays a role in regulating IRS-1 and glucose metabolism with respect to the Warburg effect. Insulin resistance causes diminished glucose uptake not only in type 2 diabetes mellitus (DM2) but also in the brain in Alzheimer’s disease (AD) [35]. IRS-1-mediated physiology is regulated by its several tyrosine residues and certain serine and threonine residues, which promote and reduce insulin signaling, respectively. Dysregulated phosphorylation of tyrosine and serine/threonine in IRS-1 may lead to the pathologic state of insulin resistance [36]. Recent studies indicate that phosphorylation of IRS-1 at S307 and/or at certain tyrosine residues can serve as a biomarker in peripheral exosomes, in both DM2 and AD patients [35, 37]. Once the effects of Axl on glucose uptake and/or glucose metabolism are established, novel strategies may be devised to target the molecules involved in these pathways, e.g., TNS2 and IRS-1, to improve the treatment for PDAC and diabetes. We further speculate that the interaction of Axl and TNS2 could be a potential therapeutic target for the management of the IRS-1-associated cancer progression and other related diseases.

## Conclusions

**Fig. 5:**
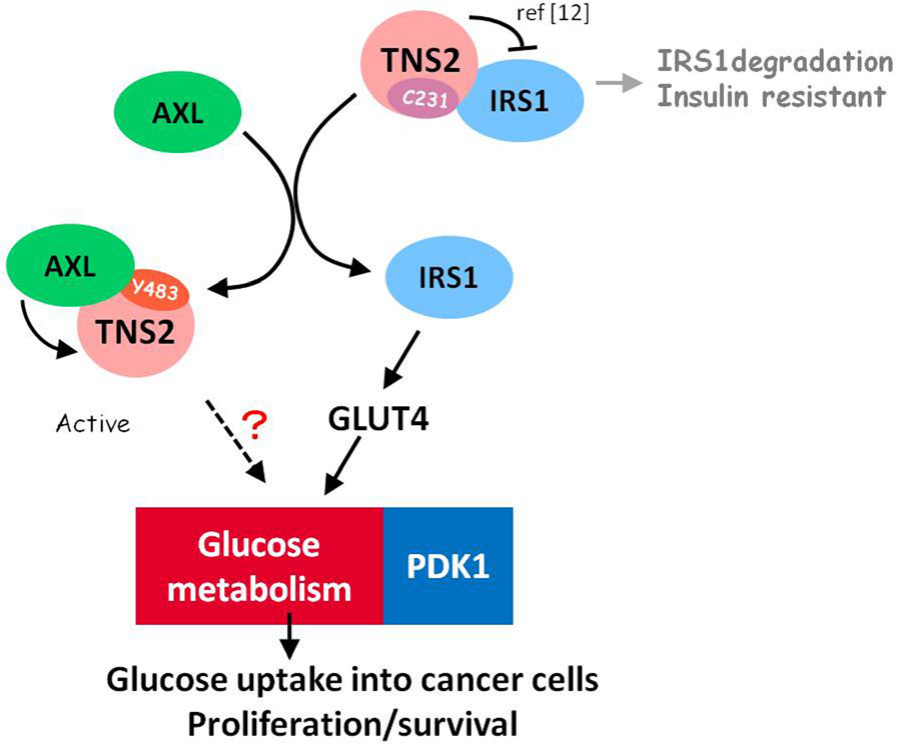
Graphical abstract. Our results revealed a novel mechanism whereby Axl binds and phosphorylates TNS2 at Y483, releasing TNS2 from interacting with IRS-1, resulting in increased stability of IRS-1. Higher levels of IRS-1 may enhance cellular glucose uptake through up-regulation of Glut 4 and cell growth and survival. The two key enzymes of aerobic glycolysis, PDK1 was found to be up-regulated by the Axl/TNS2/IRS-1 cross-talk. The details of this regulatory cross-talk remain to be elucidated

Our results revealed a novel mechanism whereby Axl binds and phosphorylates TNS2 at Y483, releasing TNS2 from interacting with IRS-1, resulting in increased stability of IRS-1 and the IRS-1 associated metabolism (Fig. 5). The interaction of Axl and TNS2 could serve as a potential therapeutic target for the management of the IRS-1-associated cancer progression and other related diseases. The Axl/TNS2/IRS-1 cross-talk may further potentially play a critical role in glucose metabolism of cancer cells.

## Additional files

Additional file 1: The endogous expression of TNS2, p-Axl and Axl in five pancreatic cancer cell lines. Densityometric quantitative analyses of the TNS2 expression and pAxl/Axl ratio was performed in bar graphs. (PDF 117 kb)

Additional file 2: The original gel image of Fig. 3a. To demonstrate the binding of TNS2 to Axl, HEK293T cells were transfected with TNS2 or variant mutants of Axl and lysed. TNS2 was co-immunoprecipitated (co-IP) by Myc and Axl. K567R: Kinase-dead Axl (KD-Axl). (PDF 132 kb)

Additional file 3: Clinicopathological characteristics of the 33 pancreatic adenocarcinoma patients. The H-score data are shown as mean ± SD. (PDF 55 kb)

Additional file 4: Total TNS2 and total AXL expression in pancreatic cancer cells. Both proteins were detected by IHC in 33 PDAC. Representative micrographs show the histopathologic features and the expressions of TNS2, Axl, IRS-1, and GLUT4 in tumor and the adjacent normal tissues by immunohistochemical analysis. (PDF 164 kb)

Additional file 5: Expression of TNS2 (TENC1) in the Oncomine reference database. (PDF 148 kb)

## Abbreviations

DLC: Deleted in liver cancer 1
FAK: Focal adhesion kinase
Gas6: Growth arrest specific protein 6
Glut4: glucose transporter type 4
IP: Immunoprecipitation
IRS-1: Insulin receptor substrate-1
KD: Kinase-dead
Mpl: Myeloproliferative leukemia
PDAC: Pancreatic ductal adenocarcinoma
PDK1: Pyruvate dehydrogenase kinase 1
PI3K: Phosphatidylinositol-3-kinase
PTB: Phosphotyrosine-binding
PTP: Protein tyrosine phosphatise
SH2: Src Homology 2
TNS2: Tensin 2
