# Supplementary figures and images for: AXL phosphorylates and up-regulates TNS2 and its implications in IRS-1-associated metabolism in cancer cells
Source: J Biomed Sci. 2018 Nov 12;25:80. doi: 10.1186/s12929-018-0465-x (PMC6233515; doi:10.1186/s12929-018-0465-x)

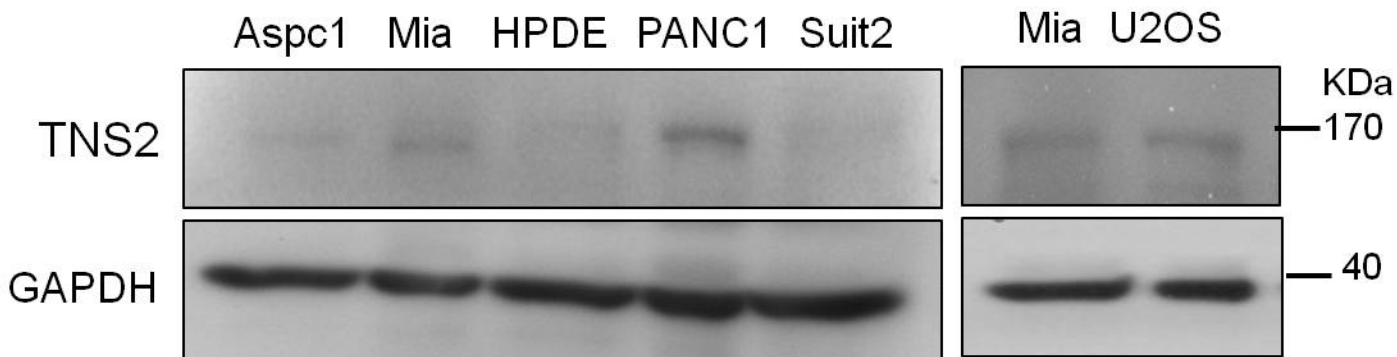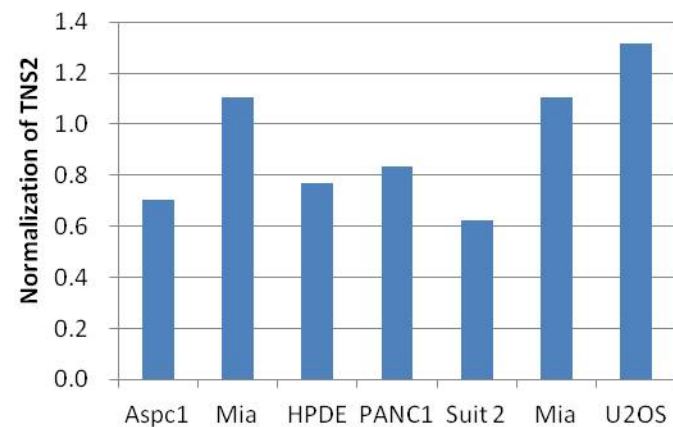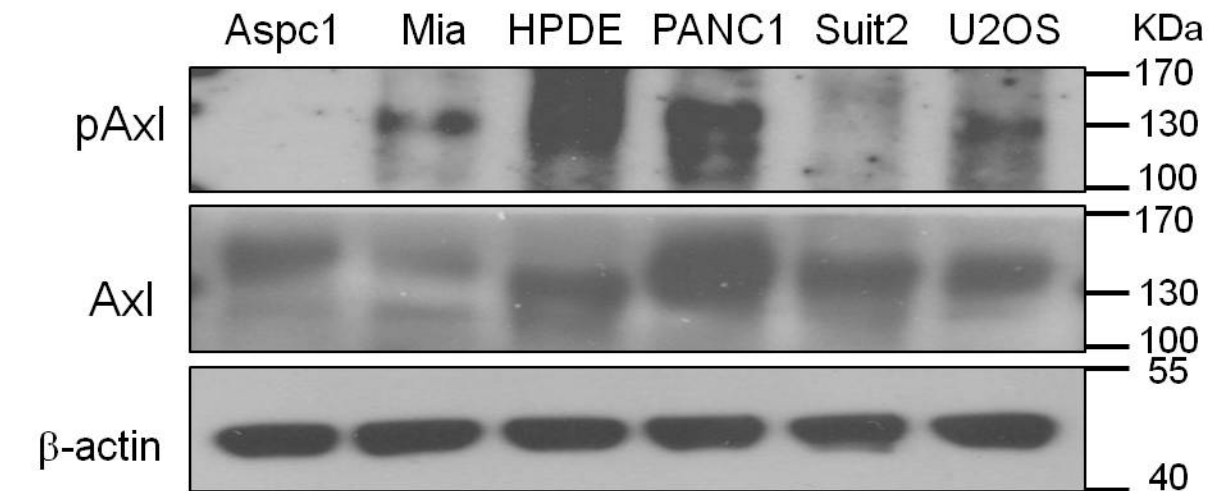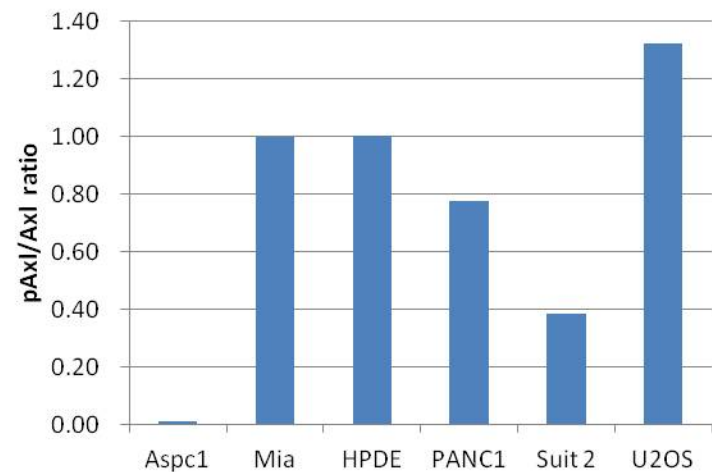

Supplement: Supplementary file 1 — The endogous expression of TNS2, p-Axl and Axl in five pancreatic cancer cell lines. Densityometric quantitative analyses of the TNS2 expression and pAxl/Axl ratio was performed in bar graphs. (PDF 117 kb) [file 12929_2018_465_MOESM1_ESM.pdf]

Additional file 4

Left--Tumor

Right--Adjacent normal

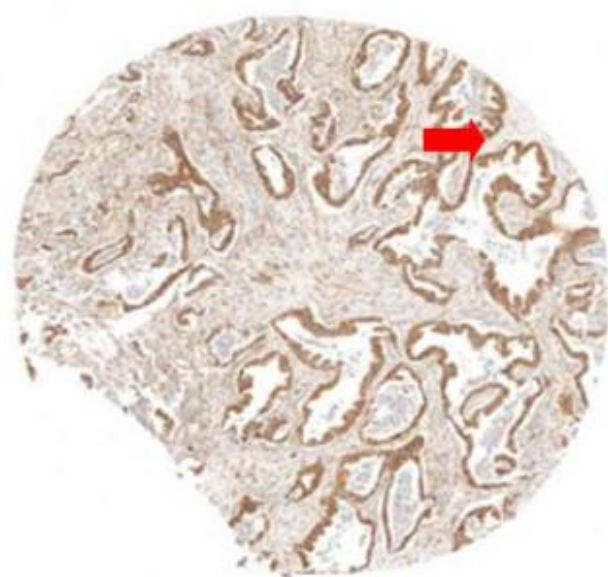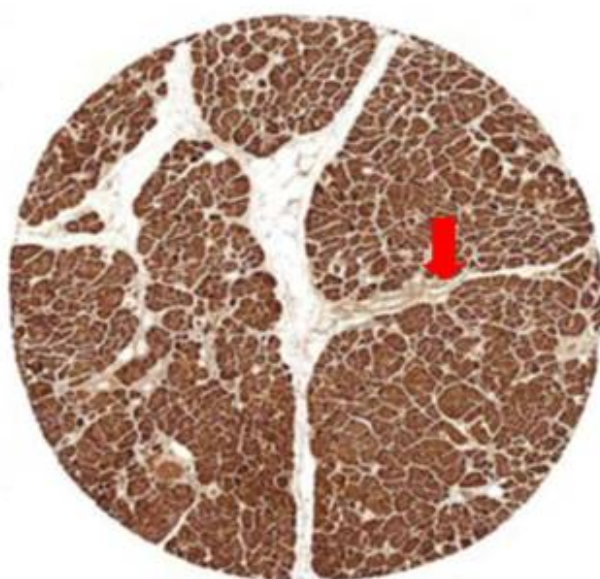

**TNS2**

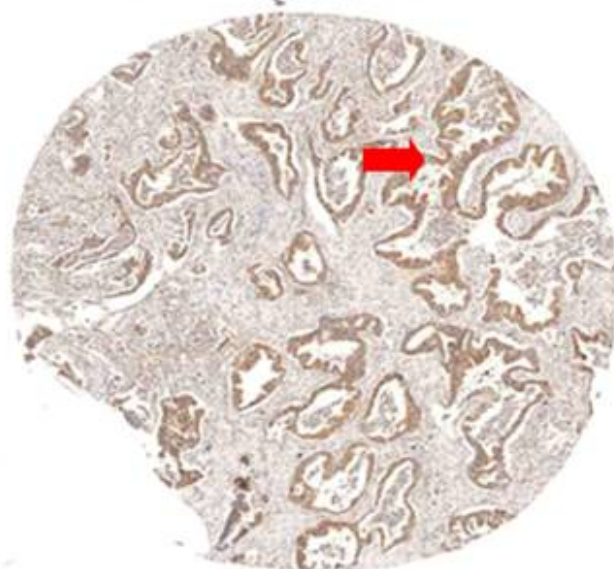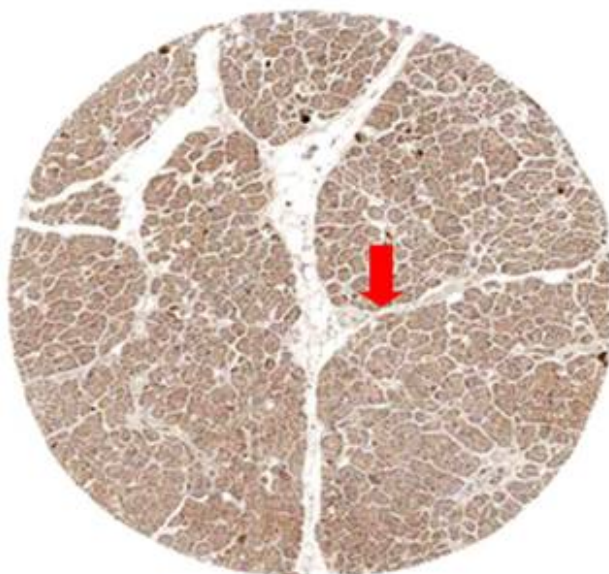

**Axl**

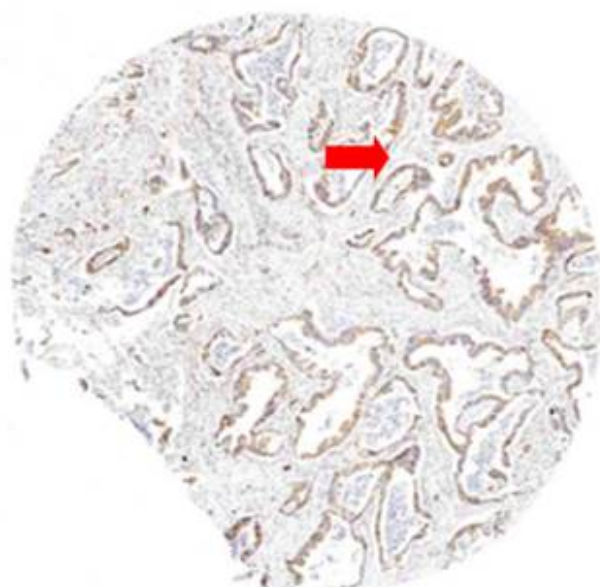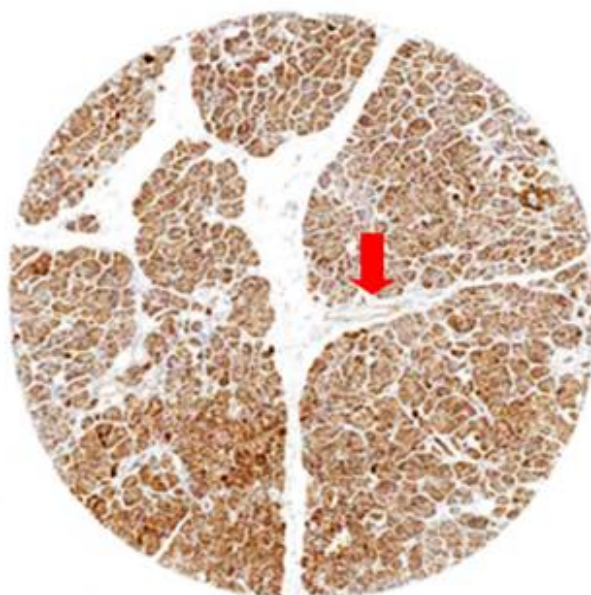

**IRS-1**

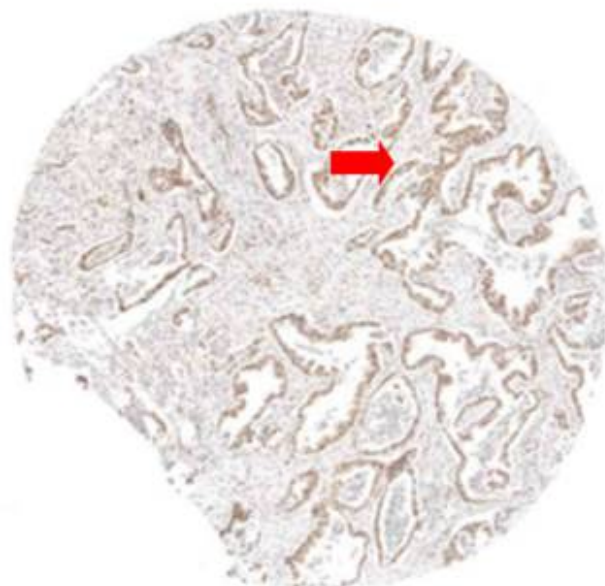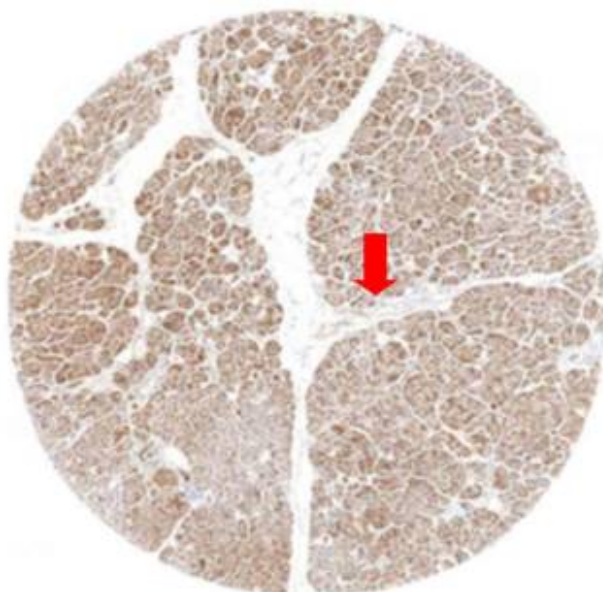

**Glut4**

Supplement: Supplementary file 4 — Total TNS2 and total AXL expression in pancreatic cancer cells. Both proteins were detected by IHC in 33 PDAC. Representative micrographs show the histopathologic features and the expressions of TNS2, Axl, IRS-1, and GLUT4 in tumor and the adjacent normal tissues by immunohistochemical analysis. (PDF 164 kb) [file 12929_2018_465_MOESM4_ESM.pdf]
