# Supplementary material for: AXL phosphorylates and up-regulates TNS2 and its implications in IRS-1-associated metabolism in cancer cells
Source: J Biomed Sci. 2018 Nov 12;25:80. doi: 10.1186/s12929-018-0465-x (PMC6233515; doi:10.1186/s12929-018-0465-x)

Additional file 2

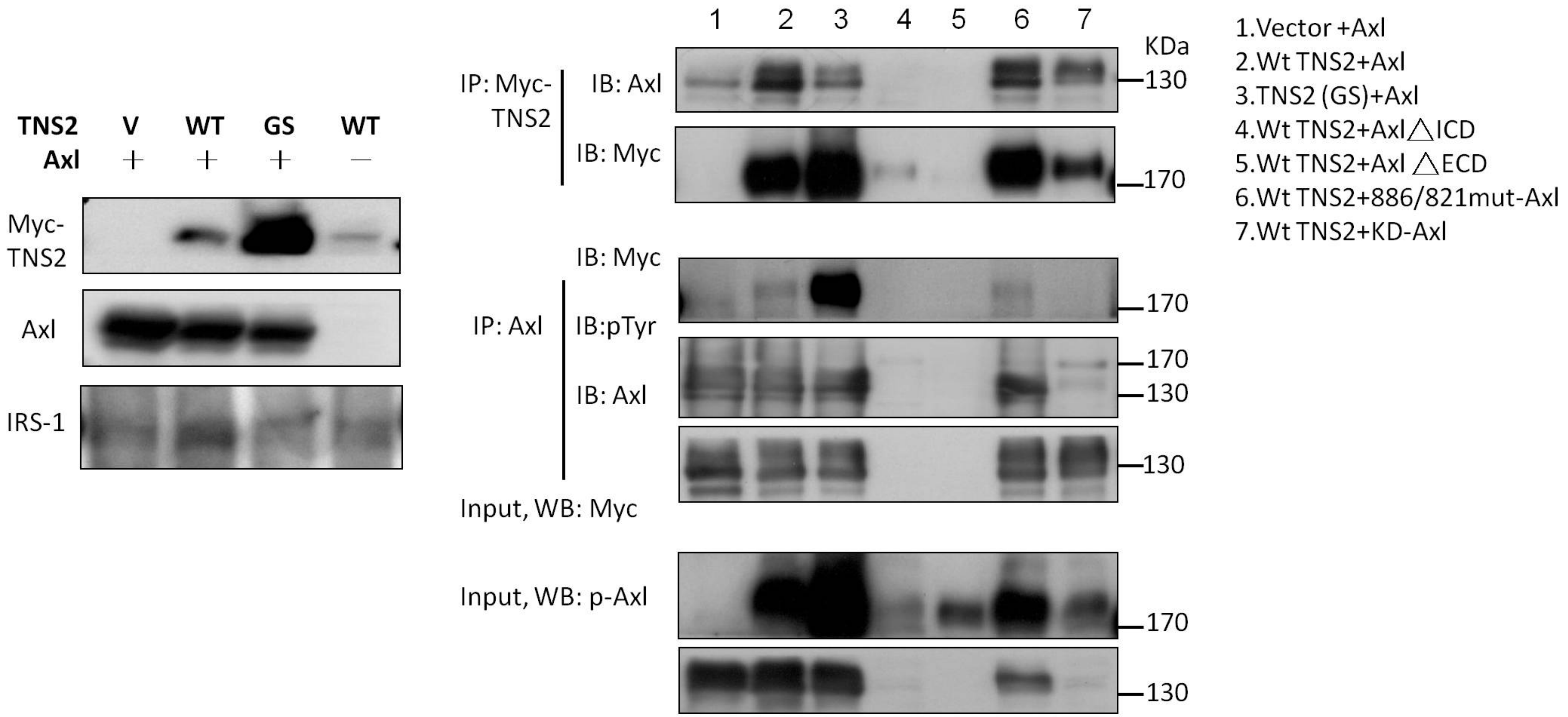

Supplement: Supplementary file 2 — The original gel image of Fig. 3a. To demonstrate the binding of TNS2 to Axl, HEK293T cells were transfected with TNS2 or variant mutants of Axl and lysed. TNS2 was co-immunoprecipitated (co-IP) by Myc and Axl. K567R: Kinase-dead Axl (KD-Axl). (PDF 132 kb) [file 12929_2018_465_MOESM2_ESM.pdf]
