# Supplementary material for: AXL phosphorylates and up-regulates TNS2 and its implications in IRS-1-associated metabolism in cancer cells
Source: J Biomed Sci. 2018 Nov 12;25:80. doi: 10.1186/s12929-018-0465-x (PMC6233515; doi:10.1186/s12929-018-0465-x)
Supplement: Supplementary file 3 — Clinicopathological characteristics of the 33 pancreatic adenocarcinoma patients. The H-score data are shown as mean ± SD. (PDF 55 kb) [file 12929_2018_465_MOESM3_ESM.pdf]

Additional file 3. Clinicopathological characteristics of the 33 pancreatic adenocarcinoma patients. Data were shown as mean ± SD of H-score.

	Early stage	Late stage
	n=17	n=16
	mean ± SD	mean ± SD
Axl	125.1 ± 30.4	134.2 ± 27.0
pAxl	93.1 ± 59.2	109.9 ± 67.9
pTNS2	151.3 ± 48.9	140.5 ± 52.2
TNS2	199.2 ± 27.6	200.2 ± 24.8
Glut4	157.6 ± 45.5	165.3 ± 35.2
IRS1	129.2 ± 45.4	129.0 ± 25.7

Early stage: stage IIa and earlier; Late stage: stage IIb and later stages
