# Supplementary material for: AXL phosphorylates and up-regulates TNS2 and its implications in IRS-1-associated metabolism in cancer cells
Source: J Biomed Sci. 2018 Nov 12;25:80. doi: 10.1186/s12929-018-0465-x (PMC6233515; doi:10.1186/s12929-018-0465-x)
Supplement: Supplementary file 5 — Expression of TNS2 (TENC1) in the Oncomine reference database. (PDF 148 kb) [file 12929_2018_465_MOESM5_ESM.pdf]

Additional file 5

Analysis Type by Cancer	Cancer vs. Normal		Cancer vs. Cancer: Cancer Histology		Cancer vs. Cancer: Multi-cancer		Clinical Outcome		Outlier	
Bladder Cancer		4				2			7	7
Brain and CNS Cancer		3	1		1				32	32
Breast Cancer	2	14	2	5	1				61	61
Cervical Cancer		1				1			8	8
Colorectal Cancer	1	13				1			31	31
Esophageal Cancer	2	3				1			10	10
Gastric Cancer		1	1	3		2			15	15
Head and Neck Cancer		5			1				22	22
Kidney Cancer	1	4	3	5	2				17	17
Leukemia			1			6			48	48
Liver Cancer		2	2	2					12	12
Lung Cancer	1	12	3	4					34	34
Lymphoma	5					8			29	29
Melanoma					4	1			20	20
Myeloma		1				1			15	15
Other Cancer	2				1				35	35
Ovarian Cancer	1	3				1			15	15
Pancreatic Cancer									13	13
Prostate Cancer									25	25
Sarcoma		6	4	4	4				23	23
Significant Unique Analyses	15	72	17	23	11	21			438	438
Total Unique Analyses	415		686		252				876	

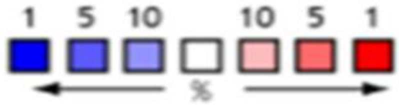

Cell color is determined by the best gene rank percentile for the analyses within the cell.
